# Supplementary material for: Mutations in FUS lead to synaptic dysregulation in ALS-iPSC derived neurons
Source: Stem Cell Reports. 2024 Jan 18;19(2):187–95. doi: 10.1016/j.stemcr.2023.12.007 (PMC10874860; doi:10.1016/j.stemcr.2023.12.007)
Supplement: Document S1. Supplemental experimental procedures, Figures S1–S3, and Table S1 [file mmc1.pdf]

Stem Cell Reports, Volume *19*

Supplemental Information

Mutations in FUS lead to synaptic dysregulation in ALS-iPSC derived neurons

Carole Shum, Erin C. Hedges, Joseph Allison, Youn-bok Lee, Natalia Arias, Graham Cocks, Siddharthan Chandran, Marc-David Ruepp, Christopher E. Shaw, and Agnes L. Nishimura

Mutations in FUS lead to synaptic dysregulation in ALS-iPSC derived neurons

Carole Shum [1, 2], Erin C. Hedges [1], Joseph Allison[1], Youn-bok Lee [1], Natalia Arias [1, 3], Graham Cocks [1], Siddharthan Chandran [4], Marc-David Ruepp [1], Christopher E. Shaw [1, 5, 8], and Agnes L. Nishimura [1, 6, 7, 8]

Author affiliations:

[1] United Kingdom Dementia Research Institute Centre, Maurice Wohl Clinical Neuroscience Institute, Institute of Psychiatry, Psychology and Neuroscience, King's College London, 5 Cutcombe Rd, SE5 9RT, London, U.K.

[2] Genetics & Genome Biology Program, The Hospital for Sick Children, Toronto, ON M5G 1X8, Canada; The Centre for Applied Genomics, The Hospital for Sick Children, Toronto, ON M5G 1X8, Canada.

[3] Department of Psychology, Faculty of Life and Natural Sciences, Brain and Behavior Group, Nebrija University, Madrid, Spain.

[4] MRC Centre for Regenerative Medicine, Euan MacDonald Centre for MND Research and Centre for Clinical Brain Sciences, University of Edinburgh, Edinburgh, EH16 4SB, UK

[5]Centre for Brain Research, University of Auckland, 85 Park Road, Grafton Auckland 1023, New Zealand

[6] Centre for Neuroscience, Surgery and Trauma, Blizard Institute, Barts and The London School of Medicine and Dentistry, Queen Mary University of London, London, UK

[7] Institute Paulo Gontijo, Brazil

Author footnotes: 8 These authors contributed equally

Contact information: Dr Agnes Nishimura (a.nishimura@qmul.ac.uk) and Prof. Christopher E. Shaw (chris.shaw@kcl.ac.uk)

Supplementary experimental procedures

Human primary cell lines

Two FUS fibroblast lines were established from a skin biopsy taken from a female carrying the R521C FUS mutation and a male patient carrying the R514G mutation. The biopsy was cut into pieces, plated in a tissue culture flask, and grown in a human fibroblast derivation medium containing DMEM, 10% fetal bovine serum, and 1% penicillin/streptomycin. The outgrowth of cells appeared after 1-2 weeks. The medium was replaced every two days, and cells were passaged 1:3 with 0.25% trypsin-EDTA upon reaching confluency. All procedures were approved by the King's College Hospital, NHS Foundation Trust ethics review committee and the Department of Research and Development.

Generation and expansion of iPSCs

Induced pluripotent stem cells were reprogrammed from dermal fibroblasts, keratinocytes and lymphoblastoid cell lines as previously reported (Bilican et al., 2012; Rajesh et al., 2011). For fibroblast-derived iPSCs, approximately 1 x 10^5 control and patient cells were plated in one well of a six-well plate and infected with the retrovirus expressing OCT4, NANOG, c-MYC and SOX2 as described previously (Barrett et al., 2014; Bilican *et al.*, 2012). Three days post-infection, cells were passaged into plates pre-seeded with inactivated mouse embryonic fibroblasts (MEFs). Seven days after infection, the medium was transitioned to human embryonic stem cell (hESC) medium (DMEM/F12 containing 20% Knockout Serum Replacement, ten ng/mL human recombinant basic fibroblast growth factor (bFGF), 1 x non-essential amino acids, 5.5 mM β-Mercaptoethanol (Sigma Aldrich) and 1 x penicillin-streptomycin), supplemented with five μM ROCK inhibitor (Y27632; Calbiochem) until visible colonies appear. Multiple colonies were collected and expanded. Lymphoblastoid derived iPSCs were generated by transfecting 1x10^6 cells with plasmids expressing OCT4, L-MYC, KLF4, SV40LT, LIN28, SOX2 and shRNA-p53 (Barrett *et al.*, 2014). Cells were plated on MEF until visible colonies appear. Colonies were manually picked and expanded. All iPSCs lines were cultured in E8 Flex media in feeder-free conditions until differentiation.

Lentiviral production

The glutamate experiments were performed in neurons transduced with FU:MAP2-GFP plasmid for better visualisation of varicose dendrites and axons. EGFP sequence was subcloned into FU-MAP2-Gateway plasmid (a gift from John Gearhart (Addgene plasmid # 43915; **http://n2t.net/addgene:43915**; RRID:Addgene_43915). Lentiviral particles were produced in HEK293FT in-house and stored at –70°C until use.

Quantitative PCR

RNA was isolated with the RNeasy kit Plus (Qiagen) according to the manufacturer's instructions. cDNA was synthesised by using 1 μg of total RNA using iScript cDNA synthesis kit (BIO-RAD) and quantitative PCR was performed using PowerUP SYBR green master mix following manufacturer's instructions. qPCR reactions were amplified in QuantStudio 7 Flex Real-Time PCR system (Applied Biosystems) and analysed in QuantStudio Real-time PCR system software (Applied Biosystems). Primer sequences for FMR1: forward: 5'-GGAACAAAGGACAGCATCGC-3'; reverse:

5’-CTCTCCAAACGCAACTGGTCT-3. Primer efficiency (E) value was calculated for FMR1 expression based on the slope of the standard curve. The E value for FMR1 expression is 2.06, and this value was incorporated in the formula:

$$RQ = \frac{E^{\Delta CT\ GOI}}{average\ ERef^{\Delta CTRef}}$$

Where:

RQ = relative quantity

E=primer efficiency

∆CT= control Ct– FUS Ct

GOI=Gene of interest

Ref=housekeeping genes

Glutamate toxicity

Neurons were plated on 96 well plates for two weeks and transduced with FU:MAP2-GFP lentivirus, following another two weeks in culture. Cells were treated with vehicle (DMSO) or 100 mM Glutamate (Abcam) for 24 hours and fixed. Cells were stained for caspase-3 antibody (BD Biosciences) and imaged using the Opera Phenix High Content Screening microscope. The total number of neurons analysed was control 1 (n=1,003), control 2 (n=1,206), R514G (n=1,634) and R521C (n=1,205).

Immunostaining

Cells were fixed with 4% paraformaldehyde or with 4% paraformaldehyde for 10 min at room temperature followed by incubation in methanol pre-chilled to -20°C for 10min, permeabilised with 0.5% Triton X-100 for 15 minutes at room temperature, then blocked in 10% donkey serum for one hour at room temperature, followed by incubation with primary and secondary (Dylight dyes) antibodies. The nuclei were counterstained with DAPI staining and coverslips mounted on slides with mounting media (DAKO).

Calcium imaging and synaptic activity analyses

Cortical neurons were plated in 96 well plates (Falcon) and aged for 108 days. Cells were incubated with 2 mM Fluo4-AM in external solution (145 mM NaCl, 2 mM KCl, 5 mM $NaHCO_3$, 1 mM $MgCl_2$, 2.5 mM $CaCl_2$, 10 mM glucose, 10mM Na-HEPES pH 7.25) or FluoroBrite DMEM media (Life Technologies) and 0.02% Pluronic-F27 for 15 mins at 37°C. Subsequently, neurons were rinsed in external solution for another 15 mins at 37°C. 80 frames were taken every second and processed in ImageJ. Spontaneous calcium fluctuation was calculated as relative Fluo4-AM fluorescence intensities compared to background: F-F0/F0. Imaging was performed in the Opera Phenix Content Screening microscope (20x water objective). 10 regions of interest (ROI) were measured and plotted on a graph to represent calcium fluctuation during time.

To determine whether calcium influx is altered upon depolarisation, 4 weeks old neurons were treated with 2 μM Fluo4-AM in FluoroBrite DMEM media and incubated for 15 mins at 37°C, followed by 15 mins wash with FluoroBrite DMEM media. Cells were imaged for 60 seconds to calculate basal fluorescence and 100 mM KCL was added to the media. Imaging recording was performed for 300 seconds. 5-10 ROIs were selected, and fluorescence was measured over time.

To measure synaptic activity, cortical neurons were cultured for 4 weeks and treated with 10 μM of FM4-64. FM4-64 incorporates into the membrane and is internalised by endocytosis when becomes fluorescent. At synapses, FM4-64 is in the synaptic vesicle membrane and after depolarisation of the membrane with KCL, the synaptic vesicles are released into the synaptic cleft, losing the fluorescence.

Western blot

Total extracts were generated from neurons by lysing the cells in 1X cold RIPA lysis buffer (50 mM Tris-HCl (pH7.4), 150mM NaCl, 1% (v/v) Triton X-100, 0.1% (w/v) sodium dodecyl sulfate (SDS), 0.1% (w/v) Sodium Deoxycholate), supplemented with 1x Proteinase inhibitor (Roche) 1x Phosphate inhibitor (Roche). Cells were incubated on ice for 30 minutes, followed by sonication for 20 seconds. Cellular lysate was centrifuged at 14,000 x g at 4°C for 20 minutes. Protein concentration was measured using DC Protein assay (BIO-RAD) and 1x SDS sample buffer (5x SDS sample buffer containing 10% (w/v) SDS, 250 mM Tris-HCl (pH 6.8), 50% (v/v) glycerol, 500mM (w/v) dithiothreitol, 0.25% (w/v) bromophenol blue) was added in each sample. Approximately 10 µg of protein was loaded in 10% NuPAGE Bis-Tris precast gels and transferred onto nitrocellulose membranes using iBlot2 Gel transfer device. Membranes were blocked with 5% BSA or non-fat milk, followed by incubation of primary antibody in 1% BSA or non-fat milk overnight at 4°C. Membranes were rinsed with TBST, followed by incubation of secondary antibodies in the same buffer. Membranes were developed using Odyssey CLx Imager (Li-CoR Biosciences).

One microliter of protein weight standards (Precision Plus Protein Dual Color Standards, BIO-RAD) was loaded in each gel for protein molecular weight estimation.

Fluorescence In situ Hybridisation

Cortical Neurons were cultured for six weeks, fixed with 4% paraformaldehyde for 15 minutes at room temperature, and pre-treated with 70% ethanol for 2 mins at -20°C. Coverslips were stored in 100% ethanol at -80°C until staining. On the day of fixing, ethanol was removed, and coverslips were incubated with 70% ethanol for 2 mins at -20°C, followed by three washes of PBS for 5 mins each.

Cells were blocked with 1% BSA in PBS and 200 mM ribonucleoside vanadyl complex (RVC) for 30 mins at room temperature. Cells were washed twice with 2× SSC (300 mM NaCl, 30 mM sodium citrate pH 7.0) and incubated in **pre-hybridization buffer** (15% formamide, 10 mM sodium phosphate, 2× SSC) for 10 min at room temperature.

Prepare 40nM *FMR1* probe in hybridisation buffer (10% dextran sulfate, 2mM RVC, 2XSCC, 10 mM sodium phosphate, 15% Formamide, 0.2% BSA 0.5 mg/ml salmon sperm) and boil at 80°C for 5 mins and transfer to ice. Add the probe to the cells and incubate at 37°C overnight in a wet chamber to avoid evaporation.

On the next day, wash cells with pre-hybridisation buffer for 30 mins at 37°C twice, followed by two washes of a solution containing 20% formamide, 2mM RVC and 0.05XSSC for 10 mins. A final wash of 2XSSC followed this for 2 mins at room temperature.

The coverslips were mounted and imaged using the INCell Analyser 6000 and Zeiss LSM 880.

The *FMR1* probe (GGACUUGUUUUUGUUUUUGUUUUGUUGCACUGAAGUUUGAUA) was obtained from Integrated DNA technologies.

GST pulldown

GST, GST-FUS wt, GST-FUS R521C, GST-FUS R514G, GST-FUS-ΔRRG3 (1-456 aa) and GST-FUS-RRG3 (423-526) constructs were generated and transformed into BL21 competent bacteria. Single bacteria clones were inoculated in Luria broth media containing 100 mg/ml ampicillin overnight at 37°C in constant agitation. The next day, 10% of a starter culture was transferred to a new tube containing Terrific broth and kept in agitation at 37°C until OD 0.6-0.8 when 0.1 mM of IPTG was added to induce expression for another 4 hours. Bacteria were pelleted at 4,000 x g for 10 mins at room temperature and frozen at -80°C until use.

Lysis buffer was added to the bacteria pellet following the MagneGST protein Purification system (Promega) instructions.

HEK cells were transfected with eGFP-FMRP construct using lipofectamine 2000. Approximately 10 mg of HEK cell lysate was incubated to 1 mg of GST construct with or without RNAse, following the MagneGST protein Purification system (Promega) instructions. The final magnetic bead was boiled in 1X laemmli buffer (BioRad) and resolved in western blots.The membranes were incubated with FUS (Proteintech) antibody and FMRP (Cell Signaling Technology).

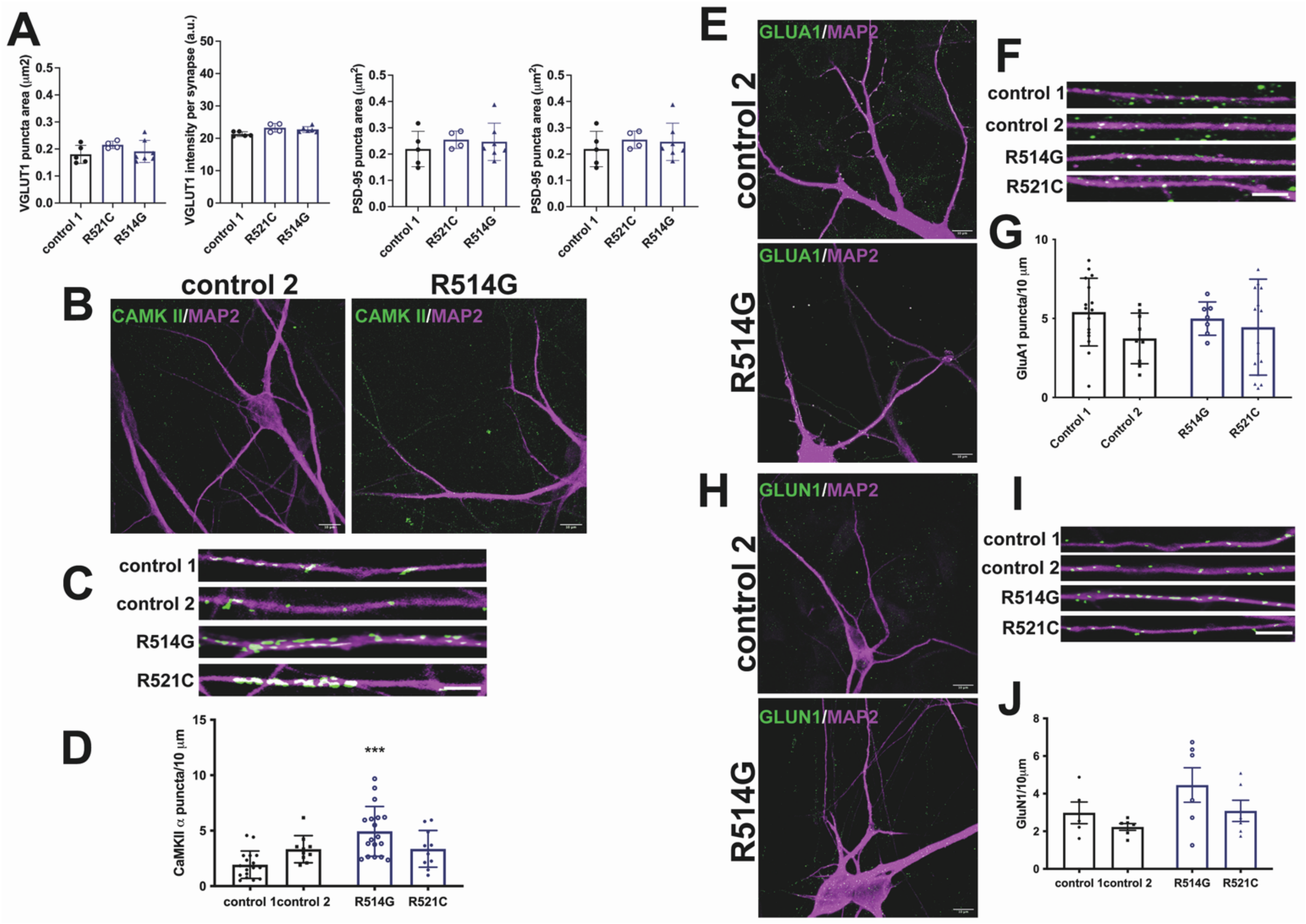

Supplementary Fig 1. **A)** Quantification of PSD-95 and VGLUT1 area and intensity from colocalised puncta. Data represent neurons analysed per group from three biological replicates**. B)** Representative confocal image of neurons expressing CAMKIIa. **C)** Representative dendrites showing CAMKIIa puncta. Scale bar = 10 μm. **D)** Quantification of CAMKIIa puncta per 10 μm dendrite. Data points represent single neurons measured as mean ± SD of 3 independent experimental replicates. **E)** Representative confocal image of neurons expressing the ionotropic glutamate receptor AMPA type GLUA1. Scale bar = 10 μm. **F)** Representative dendrites showing GLUA1 puncta. **G)** Quantification of GLUA1 puncta per 10 μm dendrite. No significant difference was observed between the two groups. Data points represent single neurons measured as mean ± SD of 3 independent experimental replicates. **H)** Representative confocal image of neurons expressing the glutamate receptor NMDA type GLUN1. **I)** Representative dendrites showing GLUN1 puncta. Scale bar = 10 μm. **J)** Quantification of GLUN1 puncta per 10 μm dendrite. Data points represent single neurons measured as mean ± SD of 3 independent experimental replicates.

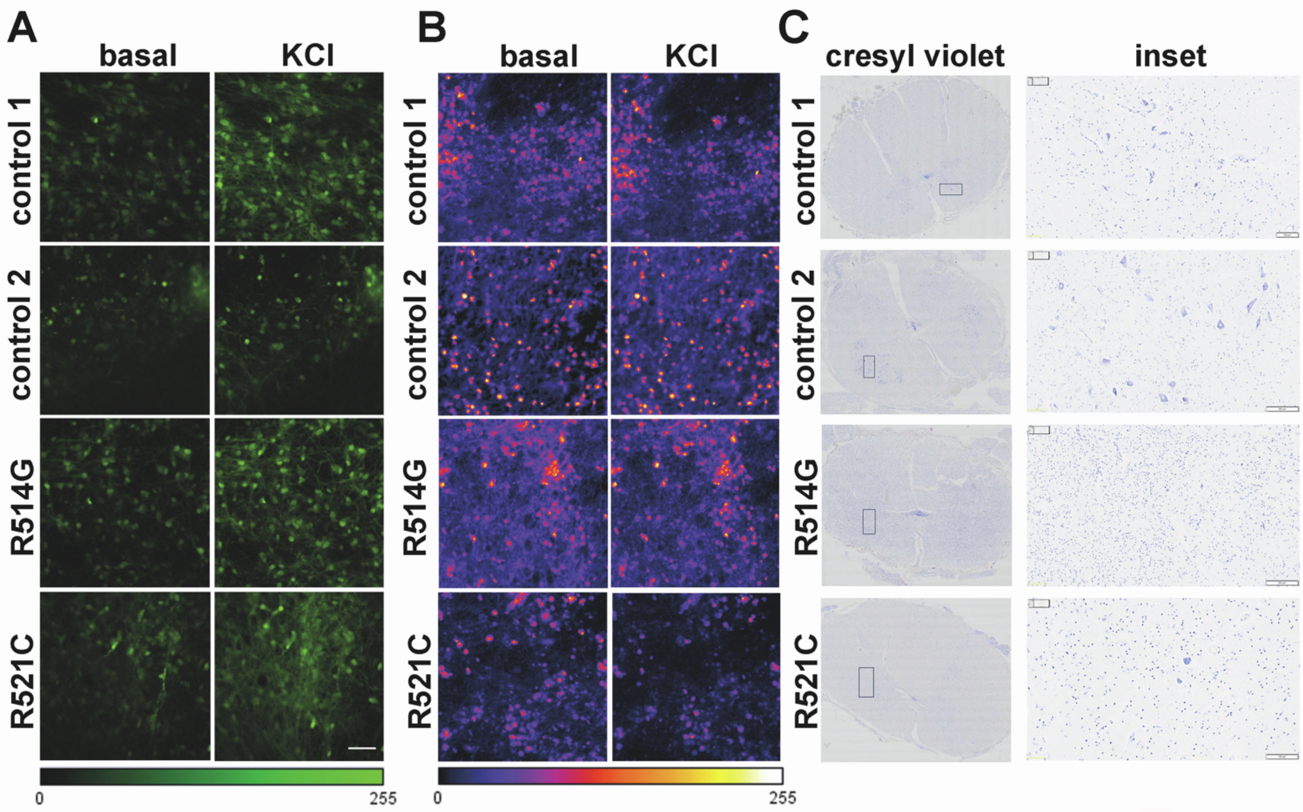

Supplementary Fig. 2. A) Representative images of neurons after Fluo-4-AM calcium staining in basal levels and after KCl depolarisation. Scale bar = 50 μm. **B)** Representative images of neurons after FM4-64 staining in basal levels and after KCl depolarisation. Scale bar = 50 μm. **C)** Cresyl Violet staining of post-mortem sections of spinal cord of control and FUS-ALS patients. A drastic reduction of motor neurons is observed in the FUS-ALS cases. Scale bar 100 μm.

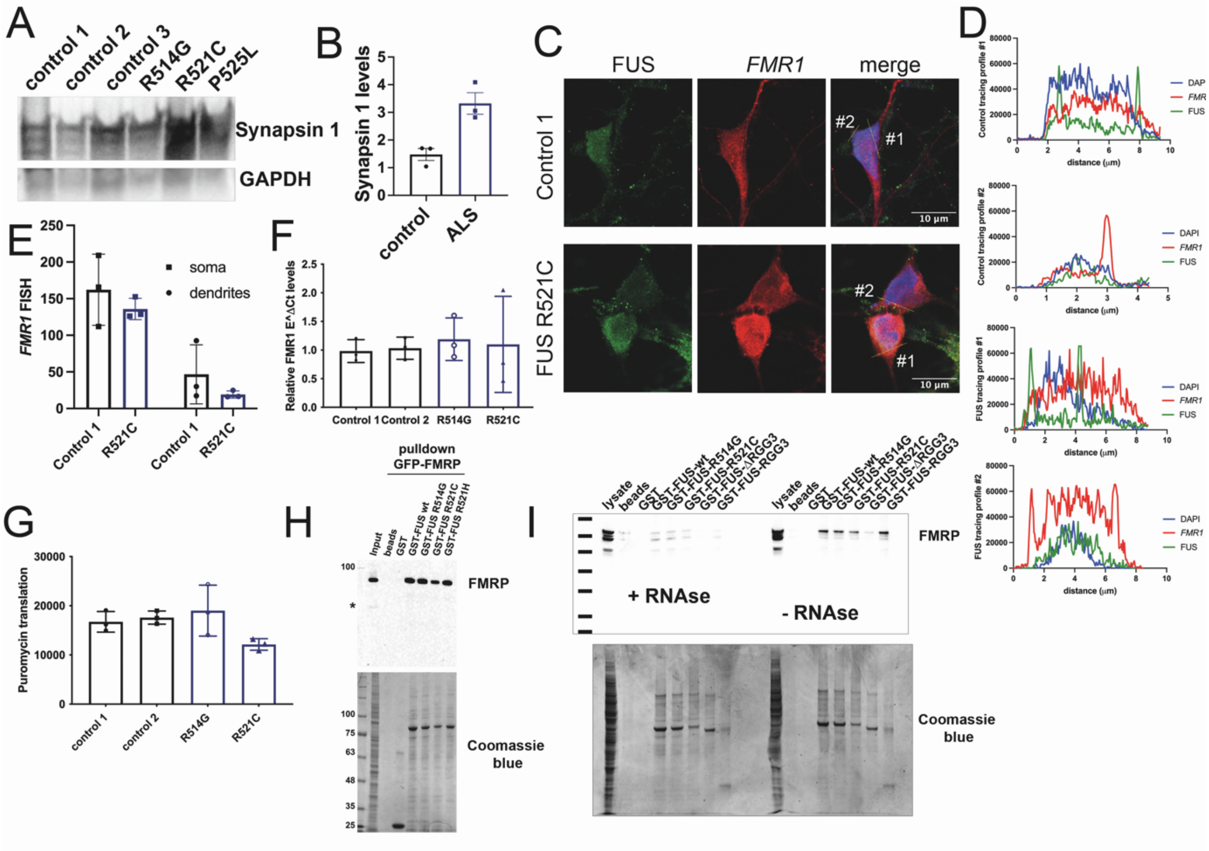

Supplementary Fig 3. A) Western blot analyses of synaptosome fraction from post-mortem cerebral cortex samples showing an increase of Synapsin 1 in FUS-ALS patients and relative expression of Synapsin 1. B) Quantification of relative Synapsin I levels as in A. C) Fluorescent In situ hybridisation (FISH) of *FMR1* mRNA indicates *FMR1* is not recruited into FUS granules in the soma or neurites. Representative images of neurons stained for FUS and *FMR1*. D) Line profile from two regions of interest. Note that FUS does not colocalise with FMR1 granules and vice-versa. A slight decrease in *FMR1* levels was observed. E) Quantification of *FMR1* FISH. F) Relative total *FMR1* q-PCR data. Data points represent three independent experiment replicates. G) Puromycin translation in FUS-ALS and control iPSC-derived neurons. Data points represent three independent experimental replicates. H) GST pulldown shows direct binding of FUS and FMRP. *Endogenous FMRP. GST proteins were incubated with eGFP-FMRP proteins and membranes were incubated with FMRP antibody. I) GST pulldown of FUS and FMRP is mediated by RNA. Note that the RGG3 domain containing the NLS regions is essential for FUS-FMRP binding.

Supplementary Table I: Clinical information for all ALS and control post-mortem spinal cord samples.

	age	sex	PMD	DIAG	Fz area	mutation
**SC-01**	60	M	66	MND	T	R514G
**SC-02**	23	F	37	MND	T	P525L
**SC-03**	39	M	51	MND	L	K510E
**SC-04**	34	F	38	MND	L	R495X
**SC-05**	35	F	19	fMND	L	R521H
**SC-06**	35	F	24	fMND	L	R521C
**SC-07**	33	M	71	fMND	L	R521C
**SC-08**	40	M	40	Control	L	-
**SC-09**	51	F	33	Control	L	-
**SC-10**	54	M	30.5	Control	L	-
**SC-11**	55	F	95	Control	L	-
**SC-14**	63	M	23	Control	L	-

References

Barrett, R., Ornelas, L., Yeager, N., Mandefro, B., Sahabian, A., Lenaeus, L., Targan, S.R., Svendsen, C.N., and Sareen, D. (2014). Reliable generation of induced pluripotent stem cells from human lymphoblastoid cell lines. Stem Cells Transl Med *3*, 1429-1434. 10.5966/sctm.2014-0121.

Bilican, B., Serio, A., Barmada, S.J., Nishimura, A.L., Sullivan, G.J., Carrasco, M., Phatnani, H.P., Puddifoot, C.A., Story, D., Fletcher, J., et al. (2012). Mutant induced pluripotent stem cell lines recapitulate aspects of TDP-43 proteinopathies and reveal cell-specific vulnerability. Proc Natl Acad Sci U S A *109*, 5803-5808. 10.1073/pnas.1202922109.

Rajesh, D., Dickerson, S.J., Yu, J., Brown, M.E., Thomson, J.A., and Seay, N.J. (2011). Human lymphoblastoid B-cell lines reprogrammed to EBV-free induced pluripotent stem cells. Blood *118*, 1797-1800. 10.1182/blood-2011-01-332064.
