# Supplementary material for: Mutations in FUS lead to synaptic dysregulation in ALS-iPSC derived neurons
Source: Stem Cell Reports. 2024 Jan 18;19(2):187–95. doi: 10.1016/j.stemcr.2023.12.007 (PMC10874860; doi:10.1016/j.stemcr.2023.12.007)
Supplement: Document S2. Article plus supplemental information [file mmc2.pdf]

Stem Cell Reports
Report
OPEN ACCESS

Mutations in FUS lead to synaptic dysregulation in ALS-iPSC derived neurons

Carole Shum,[1,2,3] Erin C. Hedges,[1] Joseph Allison,[1] Youn-bok Lee,[1] Natalia Arias,[1,4] Graham Cocks,[1] Siddharthan Chandran,[5] Marc-David Ruepp,[1] Christopher E. Shaw,[1,6,9,*] and Agnes L. Nishimura[1,7,8,9,*]
[1]United Kingdom Dementia Research Institute Centre, Maurice Wohl Clinical Neuroscience Institute, Institute of Psychiatry, Psychology and Neuroscience, King's College London, 5 Cutcombe Rd, London SE5 9RT, UK
[2]Genetics & Genome Biology Program, The Hospital for Sick Children, Toronto, ON M5G 1X8, Canada
[3]The Centre for Applied Genomics, The Hospital for Sick Children, Toronto, ON M5G 1X8, Canada
[4]Department of Psychology, Faculty of Life and Natural Sciences, Brain and Behavior Group, Nebrija University, Madrid, Spain
[5]MRC Centre for Regenerative Medicine, Euan MacDonald Centre for MND Research and Centre for Clinical Brain Sciences, University of Edinburgh, Edinburgh EH16 4SB, UK
[6]Centre for Brain Research, University of Auckland, 85 Park Road, Grafton Auckland 1023, New Zealand
[7]Centre for Neuroscience, Surgery and Trauma, Blizard Institute, Barts and The London School of Medicine and Dentistry, Queen Mary University of London, London, UK
[8]Institute Paulo Gontijo, São Paulo, Brazil
[9]These authors contributed equally
*Correspondence: chris.shaw@kcl.ac.uk (C.E.S.), a.nishimura@qmul.ac.uk (A.L.N.)
https://doi.org/10.1016/j.stemcr.2023.12.007

SUMMARY

Amyotrophic lateral sclerosis (ALS) is a fatal, adult-onset neurodegenerative disorder characterized by progressive muscular weakness due to the selective loss of motor neurons. Mutations in the gene *Fused in Sarcoma* (*FUS*) were identified as one cause of ALS. Here, we report that mutations in *FUS* lead to upregulation of synaptic proteins, increasing synaptic activity and abnormal release of vesicles at the synaptic cleft. Consequently, FUS-ALS neurons showed greater vulnerability to glutamate excitotoxicity, which raised neuronal swellings (varicose neurites) and led to neuronal death. Fragile X mental retardation protein (FMRP) is an RNA-binding protein known to regulate synaptic protein translation, and its expression is reduced in the FUS-ALS lines. Collectively, our data suggest that a reduction of FMRP levels alters the synaptic protein dynamics, leading to synaptic dysfunction and glutamate excitotoxicity. Here, we present a mechanistic hypothesis linking dysregulation of peripheral translation with synaptic vulnerability in the pathogenesis of FUS-ALS.

INTRODUCTION

Amyotrophic lateral sclerosis (ALS) is a neurodegenerative disorder characterized by progressive muscular weakness and selective motor neuron loss. Approximately 5%–10% of ALS cases have a family history of this disease caused by mutations in several genes, including *Fused in Sarcoma* (*FUS*) (Vance et al., 2009).

FUS is a ubiquitously expressed protein involved in DNA repair, transcription regulation, RNA processing, and dendritic spine morphology (Mackenzie et al., 2011; Neumann et al., 2009). It is mainly localized in the nucleus and shuttles from the nucleus to the cytoplasm. Most mutations in *FUS* are localized near the nuclear localization site (NLS) (Shang and Huang, 2016). Consequently, FUS is not effectively shuttled into the nucleus, remaining in the cytoplasm.

The exact function(s) of FUS at the synapse is (are) still unclear, and how mutant FUS induces synaptic degeneration remains to be elucidated. Previous studies have shown that FUS is involved in the spine formation (Fujii et al., 2005; Sephton et al., 2014). In FUS-overexpression animal models, synaptic dysfunction, reduced dendritic arbors, and stunted post-synapse development are observed (Ishigaki et al., 2017; Qiu et al., 2014). FUS may play a role in the local translation of synaptic proteins (Yasuda et al., 2013). Furthermore, FUS RNA targets are associated with synaptic organization and plasticity, leading to alterations in the density and size of GABAergic synapses. Further, synaptic FUS increases neuronal activity in the frontal cortex of FUS knockin mice, locomotor hyperactivity, and altered social interactions (Scekic-Zahirovic et al., 2021).

Here, we evaluate the effect of mutations in *FUS* at synapses and investigate how the increase of cytoplasmic FUS leads to synaptic dysfunction in neurons derived from ALS patients. Our results show that FUS-ALS lines increase synaptic proteins, leading to an excess of synaptic vesicle release. Consequently, neurons are more vulnerable to excitotoxicity and neuronal death.

RESULTS

ALS-linked FUS leads to synaptic protein dysregulation

Recent findings have suggested that FUS has an important role in synapses. To address whether FUS mutations lead to synaptic dysfunction, we differentiated induced pluripotent stem cells (iPSCs) from two patients carrying the R521C and R514G mutations and two controls into cortical neurons (Figure 1A). As previously shown, mutations in *FUS* disrupt the shuttling of FUS from the cytoplasmic to

This is an open access article under the CC BY license (http://creativecommons.org/licenses/by/4.0/).

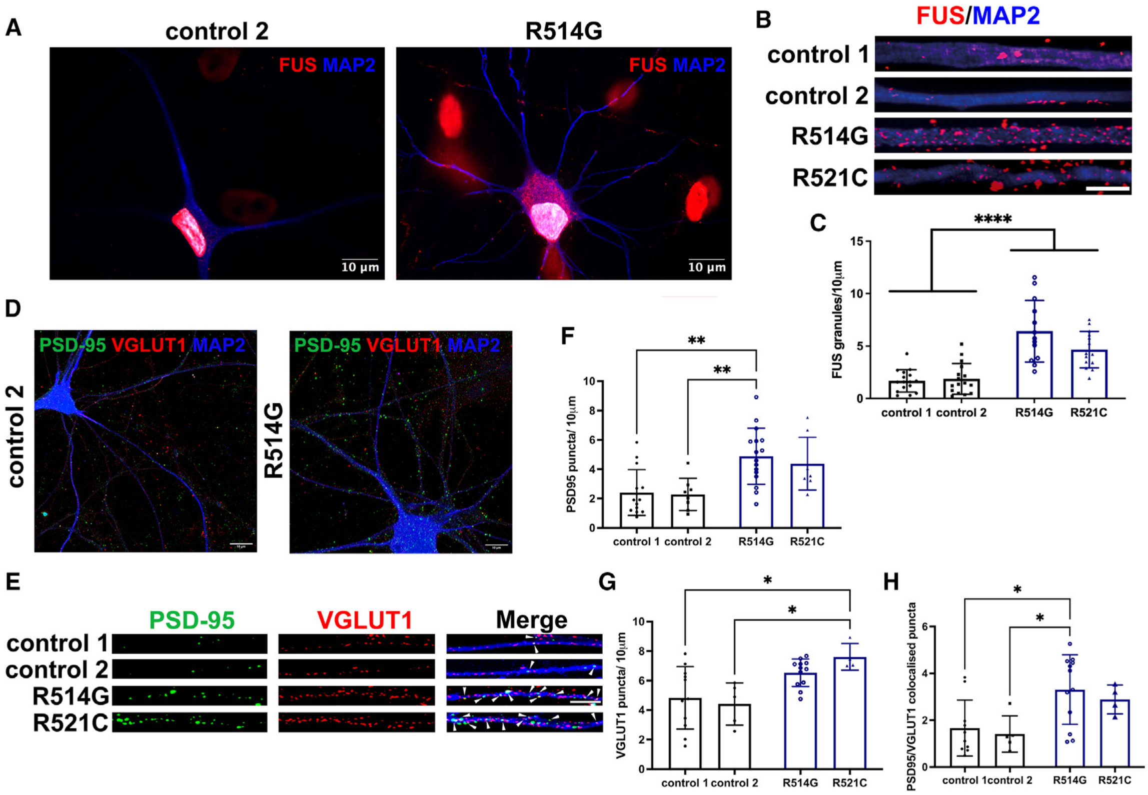

Figure 1. FUS lines show increased synaptic proteins

(A) Representative image of cortical neurons stained with MAP2 (blue) and FUS (red).

(B) Increase of FUS puncta in dendrites of FUS-ALS lines.

(C) Quantification of FUS puncta per 10-µm dendrite. Each data point represents a neuron. Three independent differentiated batches were analyzed. One-way ANOVA; ****$p < 0.0001$.

(D) Representative image of control and FUS-ALS neurons expressing PSD-95 (green), VGlut1 (red), and MAP2 (magenta).

(E) Representative dendrites showing PSD-95 and VGlut1 puncta.

(F–H) (F) Quantification of PSD-95, (G) VGlut1, and (H) co-localization of PSD-95 and VGlut1 puncta per 10-µm dendrite (arrowheads). Each data point represents a neuron. Three independent differentiated batches were analyzed. One-way ANOVA, *post hoc* Tukey analyses; *$p < 0.05$, **$p < 0.01$. Scale bar, 10 µm.

the nucleus, causing an increase of FUS in the cytoplasm and at synaptic dendrites (Figures 1A–1C). To investigate the distribution of synaptic proteins, we aged neurons for over 2 months. We observed that the FUS-ALS lines exhibited an increased density of postsynaptic density protein 95 (PSD-95) and the pre-synaptic vesicular glutamate transporter 1 (VGlut1) (Figures 1D–1G). Puncta size and intensity were assessed, and we did not find significant differences (Figure S1A). However, an increase in the PSD-95/VGlut1 colocalization was observed (Figure 1H). Our data suggest that increased synaptic puncta correspond to increased synaptogenesis.

Furthermore, the synaptic vesicle Synapsin 1 (Figures 2A–2C), gephyrin (inhibitory synapses) (Figures 2D–2F), and the calcium/calmodulin-dependent protein kinase II (CAMKIIa) (glutamatergic neurons) (Figures S1B–S1D) puncta were also increased in FUS-ALS. To determine whether the glutamate receptors are altered in the mutant lines, we quantified the AMPA receptor GLUA1 and the *N*-methyl-D-aspartate (NMDA) receptor (NMDAR) GLUN1 (Figures S1E–S1J). Our data show no significant differences in GLUN1 and GLUA1 puncta distribution between lines. We further evaluated whether increased synaptic proteins impair synaptic transmission in the FUS-ALS lines. Neurons were stained with Fluo4-AM, a dye with high calcium-binding affinity, which acts as a calcium indicator. Fluo4-AM enters the cells through endocytosis, and no obvious changes were observed in the endocytic rate (Figure 2G). Next, we measured calcium fluctuation (Fluo4-AM activity) in mature neurons under basal conditions (Figure 2H). Calcium intake is an indirect measure of synaptic activity. Therefore, we assessed the consequences of an increase in

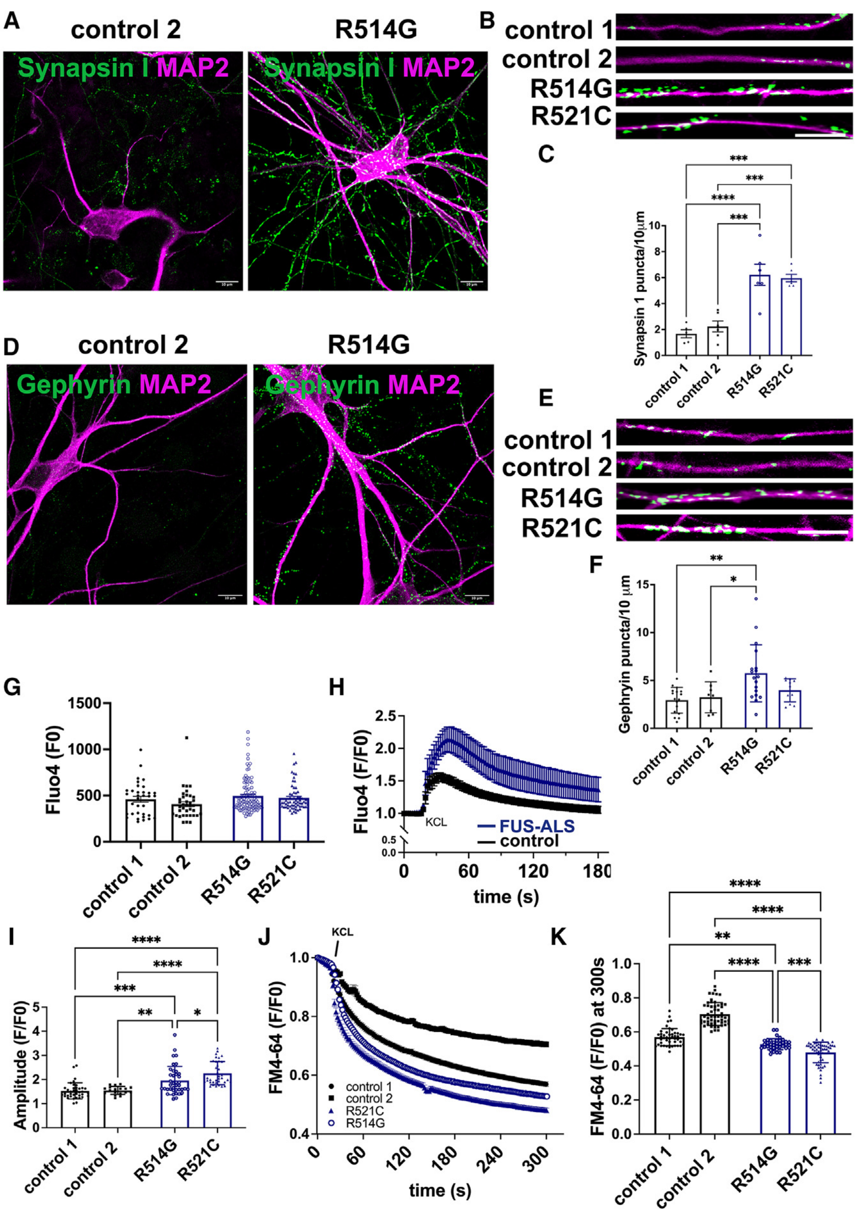

Figure 2. FUS lines present increased synaptic puncta

(A) Representative confocal image of neurons expressing Synapsin 1.

(B) Representative dendrites showing Synapsin 1 puncta.

(C) Quantification of Synapsin 1 puncta per 10-μm dendrite. Each data point represents a neuron. Three independent differentiated batches were analyzed. One-way ANOVA, *post hoc* Tukey analyses; ***$p < 0.001$, ****$p < 0.0001$.

(D) Representative image of neurons expressing gephyrin 1.

(E) Representative dendrites showing gephyrin 1 puncta.

(F) Quantification of gephyrin 1 puncta per 10-μm dendrite. Each data point represents a neuron. Three independent differentiated batches were analyzed. One-way ANOVA, *post hoc* Tukey analyses; *$p < 0.05$, **$p < 0.01$. Scale bar, 10 μm.

(G) Calcium imaging of cortical neurons derived from FUS-ALS iPSCs loaded with Fluo4-AM at F0 t0. No obvious changes are observed. Each data point represents a neuron. Three independent differentiated batches were analyzed.

(H) Calcium levels (Fluo4-AM) upon KCl depolarization over time.

(I) Peak values of calcium influx (amplitude) (one-way ANOVA, *post hoc* Tukey analyses; *$p < 0.05$, **$p < 0.01$, ***$p < 0.001$, ****$p < 0.0001$). Data are represented as mean ± SD of three independent experiments.

(J) FM4-64 fluorescence variation upon KCl depolarization over 300 s. Data are represented as mean ± SD of three independent experiments.

(K) FM4-64 fluorescence levels at t = 300 s (one-way ANOVA, *post hoc* Tukey analyses; **$p < 0.01$, ***$p < 0.001$, ****$p < 0.0001$). Each data point represents a neuron. Three independent differentiated batches were analyzed.

synaptic proteins when neurons are depolarized. Neurons stained with Fluo4-AM were treated with 100 mM KCl to simulate depolarization. KCl reliably depolarizes neurons by allowing calcium influx, thus increasing the fluorescence signal. As expected, FUS-ALS lines showed a more extensive and prolonged fluorescence response to depolarization than control lines (Figures 2H and S2A) and a higher fluorescence peak (amplitude) (Figures 2I and S2A). The increase of Synapsin 1 indicates more synaptic vesicles. To study synaptic activity, we used the fluorescent dye FM4-64, which integrates into the synaptic vesicle membranes. After inducing depolarization using KCl, the fluorescence signal decreased, suggesting that synaptic vesicles were released. FUS-ALS neurons showed a faster and more significant reduction of FM4-64 fluorescence than controls, indicating higher levels of synaptic vesicle release (Figures 2J and S2B). Furthermore, fluorescence levels at the endpoint (t = 300 s) were significantly reduced in the mutant FUS lines (Figure 2K).

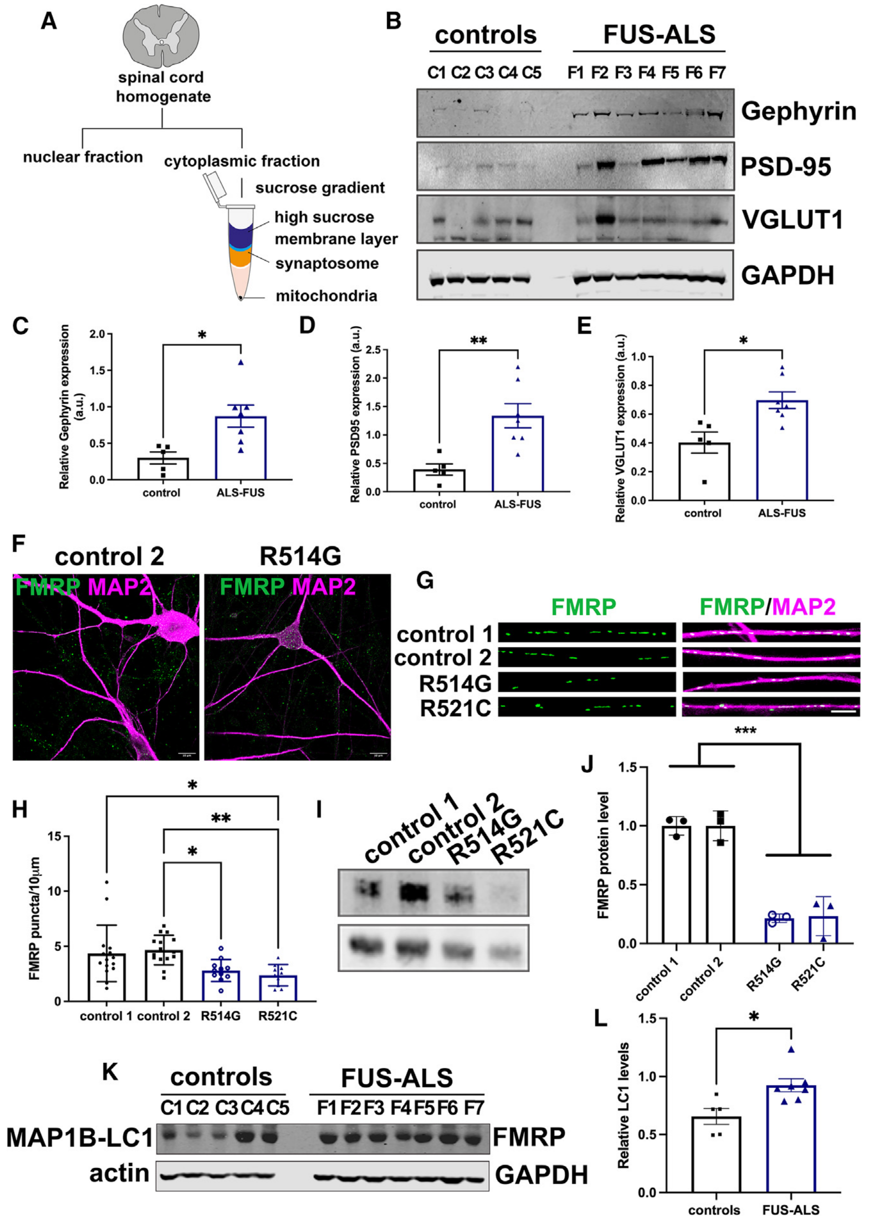

Figure 3. Synaptic profile in *post mortem* spinal cord samples and decrease of FMRP in FUS-ALS lines

(A) Schematic representation of synaptosome fractionation from spinal cord tissue.

(B) Western blot analyses of synaptosome fraction show an increase of synaptic proteins in FUS-ALS lines. FUS-ALS patients harbored the following mutations in the FUS gene: F1 = R521C; F2 = R521C, F3 = R521H, F4 = R495X, F5 = K510E, F6 = P525L, F7 = R514G.

(C) Relative expression of gephyrin.

(D) Relative expression of PSD-95.

(E) Relative expression of VGlut1. Each data point of (C)–(E) represents a control or a patient pooled together in one independent experiment. Data are represented as mean ± SEM. T-test analyses, with Bonferroni correction. *$p < 0.05$, **$p < 0.01$.

(F) Representative confocal image of control and FUS-ALS neurons expressing FMRP. Scale bar, 50 μm.

(G) Representative dendrites showing FMRP puncta. Scale bar, 10 μm.

(H) Quantification of FMRP puncta per 10-μm dendrite. Each data point represents one neuron. Three independent differentiated batches were analyzed. One-way ANOVA, *post hoc* Tukey analyses; *$p < 0.05$, **$p < 0.01$.

(I) Western blotting of FMRP levels in iPSC-derived neurons.

(J) Quantification of FMRP levels in iPSC-derived neurons from controls and FUS-ALS lines. Each data point represents the mean ± SD of three independent experiments. One-way ANOVA; ***$p < 0.001$.

(K) FMRP target MAP1B-LC1 is increased in FUS-ALS spinal cord samples.

(L) Quantification of relative MAP1B-LC1 protein levels. Each data point represents a control or a patient. Data are represented as mean ± SEM of one experiment; t test with Bonferroni correction (*$p < 0.05$).

To validate our results in FUS-ALS *post mortem* samples, we isolated synaptoneurosome complexes from controls and FUS-ALS patients' spinal cord samples (Figure 3A; Table S1). Corroborating our results, we observed an increase of synaptic proteins in the synaptoneurosome fractions of FUS-ALS patients' samples (Figure 3). Due to the limited amount of material recovered from this experiment, we only detected strong signals from gephyrin, PSD-95, and VGlut1 (Figures 3B–3E). Notably, motor neurons are reduced in ALS (Figure S2C), indicating the synaptoneurosome fraction contains other neuronal populations. Unfortunately, we were unable to perform staining of the synaptic proteins in other neurons found in the spinal cord samples. Furthermore, the synaptoneurosome fractionation was repeated using samples from the cortex, obtaining similar results (Figures S3A and S3B).

Our results suggest that synaptic FUS may influence synaptic activity by regulating other synaptic proteins in iPSC-derived neurons and *post mortem* samples.

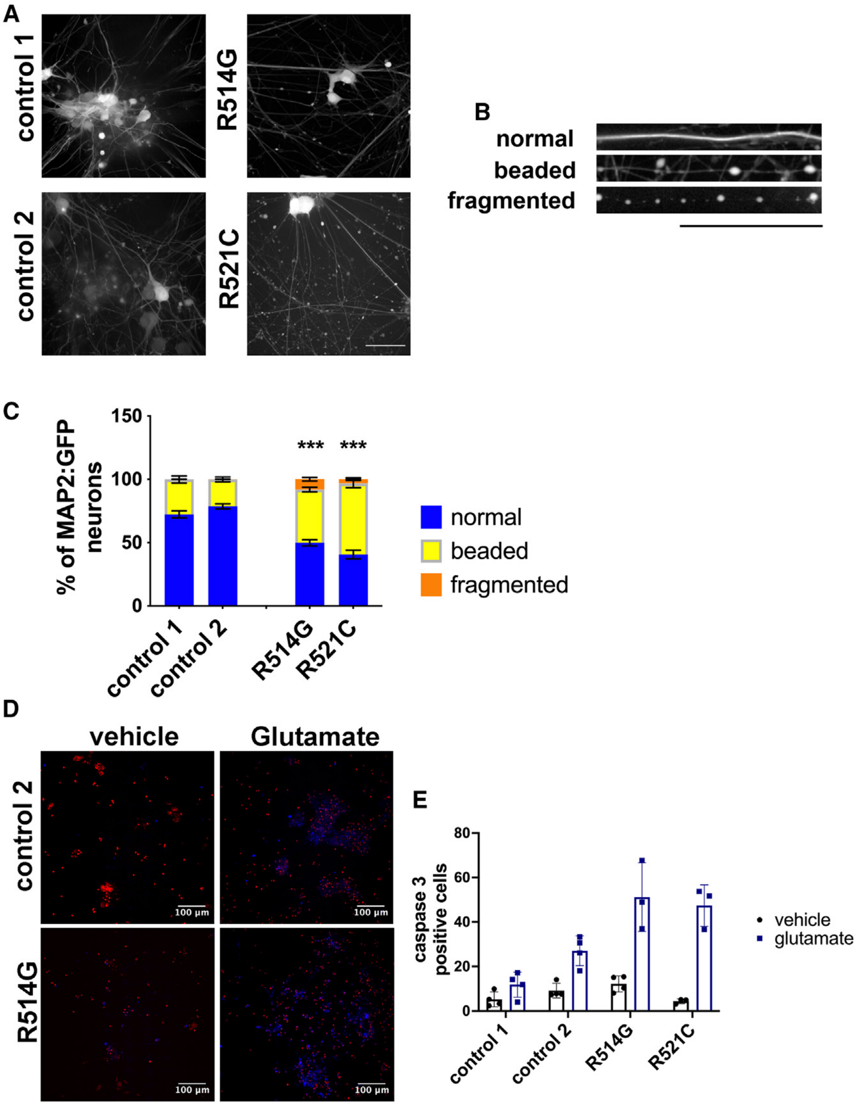

Figure 4. FUS-ALS lines are more vulnerable to glutamate excitotoxicity

(A) Representative neurons transduced with EGFP under MAP2 promoter in neuron differentiation culture. FUS neurons show an increase in neurite swellings/beading. Scale bar, 50 µm.

(B) Visual classification of normal, beaded, and fragmented neurites. Scale bar, 50 µm.

(C) Quantification of neurons containing neurite swellings as classification in (B). Data are represented as mean ± SEM of four independent experiments. One-way ANOVA; ***$p < 0.001$.

(D) Representative image of neurons treated with vehicle or 100 mM glutamate. Cells were stained with active caspase 3 (blue) and propidium iodide (red) after 24 h.

(E) Increased neuronal death in FUS lines treated with 100 mM glutamate for 24 h. Neurons expressing active caspase 3 were quantified. Data are represented as mean ± SD of three independent experiments. One-way ANOVA, *post hoc* Tukey analyses; *$p < 0.05$.

FUS interacts with the fragile X mental retardation protein (FMRP) (He and Ge, 2017), which is localized at synapses and regulates local protein synthesis (He and Ge, 2017). To investigate whether FMRP is dysregulated in FUS-ALS, we quantified FMRP puncta distribution at dendrites (Figures 3F–3H). In contrast to other synaptic proteins, FMRP puncta were reduced at the dendrites of FUS-ALS and showed an overall decrease in total protein levels by western blot (Figures 3I and 3J). We also investigated whether FUS recruits *FMR1* mRNA by fluorescent *in situ* hybridization and co-stained with FUS (Figures S3C and S3D). No changes in the localization of *FMR1* were observed. However, *FMR1* is slightly decreased in the mutant line compared to the control without reaching statistical significance (Figure S3E). Further, we measured total *FMR1* mRNA expression by qRT-PCR. No significant differences were observed (Figure S3F). Collectively, our data suggest that FMRP reduction may occur at the translational or post-translational level. Unfortunately, FMRP was undetectable in the synaptoneurosome experiments, which is probably due to the low abundance of the protein at this fraction. To confirm the effect of FMRP reduction in neurons, we investigated the well-characterized FMRP target for translational suppression, the microtubule-associated protein MAP1B-LC1. FMRP directly binds to MAP1B-LC1, regulating its expression. We assessed the MAP1B-LC1 levels in the *post mortem* samples, and we observed an increase of this protein in the synaptosome fraction of FUS-ALS patients compared to controls (Figures 3K and 3L). This indicates that defects in FMRP regulation may lead to dysregulation of synaptic proteins in FUS-ALS lines. We also investigated whether defects in protein translation

cause upregulation of synaptic proteins using puromycin-labeled conditions (Figure S3G). Puromycin levels were reduced in the R521C FUS-ALS line. However, the R514G line shows no changes in the puromycin translation.

To confirm that FUS directly binds to FMRP, we conducted glutathione S-transferase (GST) pull-down experiments (Figure S3H). GST-FUS wild type (WT), R521C, and R514G bind to FMRP, with stronger binding in the presence of RNA (Figure S3I). Furthermore, the RRG3 domain, which contains the NLS, is pivotal in FUS-FMRP binding. Deleting the RGG3 domain reduces FUS-FMRP binding, whereas the RGG3 domain alone mediates FUS-FMRP binding (Figure S3I).

FUS-ALS neurons show increased neuronal beading and vulnerability to glutamate toxicity

Next, we investigated whether increased neurotransmission leads to excitotoxicity. Mutant lines presented an increase of bead-like structures in the neurites, also known as neuritic swellings (Figure 4A). To visualize the presence of neuritic swellings, neurons were transduced with lentiviral particles expressing EGFP under the neuronal MAP2 promoter (MAP2:EGFP). Neurons were classified according to the extent of focal swellings as normal, beaded, or fragmented neurites (Figure 4B). Overall, both FUS-ALS lines presented more neurons containing neuritic swellings and fragmented neurites than controls (Figure 4C). To investigate whether glutamate increases neuronal vulnerability, we treated neurons with 100 mM glutamate for 24 h. Approximately 50% of FUS-ALS cells were positive for active caspase 3 compared to 20% in control neurons (Figures 4D and 4E). Hence, FUS-ALS neurons are more vulnerable to glutamate excitotoxicity.

DISCUSSION

In this study, we observed an increase in pre and postsynaptic proteins, leading to increased synaptogenesis and synaptic transmission. We also showed that the NMDAR and AMPA glutamate receptors remained unchanged in the FUS-ALS lines, suggesting an accumulation of glutamate at the synaptic cleft. FUS-ALS are more vulnerable to glutamate excitotoxicity, which triggers a cascade of harmful biochemical events leading to neuronal death, as observed by increased active caspase 3. While several synaptic proteins are upregulated in the FUS-ALS lines, FMRP is reduced, suggesting a potential involvement of FMRP (and FUS) in regulating the abundance and function of synaptic proteins. This mechanism needs to be further explored in the future.

FMRP is a nucleo-cytoplasmic shuttling protein abundant at synapses and is the product of the *FMR1* gene (Jin and Warren, 2000). Expansion of CGG repeats in the 5′ UTR of *FMR1* results in abnormal DNA methylation and FMRP protein silencing, leading to fragile X syndrome (FXS) (Devys et al., 1993). FMRP is an RNA-binding protein and interacts directly with FUS (He and Ge, 2017) and G quadruplexes (Blice-Baum and Mihailescu, 2014). FMRP regulates MAP1B expression, influencing microtubule stability and dynamics, axonal transport, growth cone motility, and synaptic function (Barnat et al., 2016; Riederer, 2007). Furthermore, FMRP associates with polyribosomes and is a known translation repressor, regulating the translation of specific mRNAs at dendritic spines of neurons (Ceman et al., 2003; Greenough et al., 2001). Thus, it has been suggested that FMRP regulates the local protein translation (Hafner et al., 2019). Despite reduced expression of FMRP at synapses, our puromycin translation assay did not support the hypothesis of increased protein translation capabilities in the FUS-ALS lines at synapses. Furthermore, Salam et al., (2021) observed that overexpression of mutant FUS R514G alters synaptic number in primary neurons, accompanied by increased global protein translation (Salam et al., 2021). Our data show that R514G does not enhance global puromycin translation and differs from R521C iPSC-derived neurons. The local protein translation of synaptic proteins in FUS-ALS lines will need to be explored in future experiments.

FMRP binds to RNA as observed by individual-nucleotide resolution UV crosslinking and immunoprecipitation (iCLIP) experiments performed in mouse brains (Darnell and Klann, 2013; Darnell et al., 2011). Approximately 30% of these targets are involved in synaptic transmission, including *mGLUR5*, *GLUN1*, *PSD-95*, *Synapsin 1*, and *CAMKIIa*. Other targets include *Ubiquilin1* and *2*, *senataxin* and *alsin*, APC-RNPs (associated with cell protrusions and FUS), and the most studied FMRP target, *MAP1B-LC1* (Darnell and Klann, 2013; Darnell et al., 2011). Moreover, *Fmr1* knockout (KO) mouse models exhibit exacerbated synthesis of specific proteins such as MAP1B-LC1, PSD-95, and CAMKIIa and display synaptic dysfunction caused by dysregulated local translation (Muddashetty et al., 2007; Telias, 2019).

It is still unclear how cytoplasmic FUS leads to FMRP dysregulation. One possibility is that mutant FUS decreases the protein stability of FMRP or sequesters it, reducing its protein expression or function in neurons (Birsa et al., 2021). Alternatively, cytoplasmic FUS may bind to *FMR1* mRNA directly or to an intermediate mRNA or protein, which modulates FMRP expression. Alternatively, FUS binds to selective mRNAs at synapses, including *GluN1*, *GluA1*, *CAMKIIA* (Sahadevan et al., 2021), and mRNA, which forms G-quadruplex complexes, including PSD-95 and Shank 1 (Imperatore et al., 2020). The increase in synaptic FUS levels combined with selective binding of FUS to specific synaptic mRNAs may result in a competition between

FUS and FMRP (Garone et al., 2021), displacing the latter and affecting local translation of synaptic proteins.

Scekic-Zahirovic et al. observed that the FUS$^{\Delta NLS/+}$ mouse model exhibited altered synaptic gene expression with reduced synaptic proteins, including gephyrin. This contrasts with our findings. In our study, we utilized human cellular models to study the impact of FUS on synaptic dysfunction and differentiated the cells into cortical neuron fates. It is possible that more rigorous characterization of other neuronal populations could lead to different outcomes. Further, we modeled different mutations. The FUS $^{\Delta NLS/+}$ mouse model lacks the entire exon 15 contained within the RGG3 domain. As observed in Figure S3I, FMRP binds to the RGG3 region, and deletion of the RGG3 decreases FUS-FMRP binding. Hence, we conclude that different mutations in FUS may lead to synaptic alterations caused by distinct mechanisms.

Our study has some limitations. Although we confirmed the upregulation of synaptic proteins in *post mortem* tissues, we could not detect FUS or FMRP in the synaptoneurosome fraction. Moreover, *post mortem* samples reflect an advanced stage of the disease, with increased motor neuron death and glial activation. As the samples were dissociated in bulk, we could not determine which cell populations exhibited increased synaptic proteins. Moreover, we should consider that cell culture characterization and biases in long-term cultures *in vitro* may affect the results.

In summary, our data indicate that the mislocalization of mutant FUS leads to a cascade of harmful events leading to neurodegeneration. It also suggests a common mechanism between ALS and FXS through decreased FMRP protein levels.

Our work highlights the importance of using patients' derived cells and aids understanding of the molecular events leading to neurodegeneration. Further investigation will be necessary to unravel the molecular mechanisms underpinning the involvement of FMRP in FUS-ALS neurodegeneration. Finally, the involvement of FMRP in ALS-linked neurodegeneration may shed light on potential therapeutic strategies for ALS.

EXPERIMENTAL PROCEDURES

Resource availability

Corresponding author

Further information and requests for resources should be directed to the corresponding author, Dr. Agnes Nishimura (a.nishimura@qmul.ac.uk).

Materials availability

Materials and additional details can be made available by the corresponding author upon reasonable request.

Data and code availability

No standardized datasets or new codes were generated in this study.

Derivation of cortical neurons from iPSCs

Neural induction of iPSC lines was performed using a validated protocol. Briefly, iPSCs were differentiated into neuroectoderm cells by dual SMAD signaling inhibition in induction medium (DMEM:F12 and Neurobasal [1:1], 0.5% N2 and B27, 1% Glutamax, 10 μM SB431542 [Tocris], 2.5 μM dorsomorphin [Calbiochem], 1 μM CHIR9902, and 0.2 mM ascorbic acid) for 5–7 days. Neuroepithelial cells were cultured in medium supplemented with 0.1 μM retinoic acid (Sigma-Aldrich) for 7 days. Cortical neurons were cultured in BrainPhys medium (Stemcell Technologies) containing 1% N2 and B27 and 10 ng/mL of brain-derived neurotrophic factor (BDNF) and glial cell-derived neurotrophic factor (GDNF) (R&D Systems).

Imaging analyses

Neurons were imaged with a Leica TCS-SP5 laser scanning confocal microscope (×63 oil objective, 2.5× zoom) and Nikon spinning disk confocal microscope (×100 oil objective). Quantification of neurite protrusions was performed with ImageJ plug-in NeuronJ. Fields based on uniform MAP2 staining were selected and imaged in two channels. Images were first converted to gray-scale 8 bit. Dendrites were traced, and the length of dendrites was measured using MAP2 images. The density of protrusions was measured by manually counting the number of clearly evident protrusions on primary neurites. Protrusions between 0.5 and 0.8 μm were included in the analysis.

Quantification of synaptic puncta was performed with ImageJ. Portions of MAP2-positive dendrites of at least 50 μm in length were selected. A threshold was set to capture all clusters of interest in the images containing the synaptic protein staining. The ImageJ plug-in "analyse particles" was used to measure clusters with a size of 0.08–2.5 μm^2.

Synaptoneurosoma fractionation

Post mortem spinal cord samples donated from controls and ALS cases carrying mutations in the *FUS* gene were obtained from Brain Bank, King's College London (Table S1).

Approximately 100 mg of spinal cord *post mortem* material were homogenized using a tube pestle with 10 strokes in sucrose buffer (0.32 M sucrose, 0.5 mM HEPES [pH = 7.4], 1× phoSTOP, and 1× protease inhibitor) and centrifuged at 1,000 × *g* for 10 min. The supernatant (S1) was transferred into a clean tube and centrifuged at 12,000 × *g* for 20 min at 4°C. The pellet (P2) was resuspended in sucrose buffer and transferred into a layer of sucrose gradient (1.2 and 0.8 M sucrose + 5 mM HEPES) and centrifuged at 50,000 × *g* for 1 h at 4°C.

The top layer and membrane layer were removed. Synaptosome fraction was collected and transferred to a clean tube and centrifuged at 50,000 × *g* for 2 h. The pellet containing the mitochondria fraction (pellet 3) was suspended in radioimmunoprecipitation assay (RIPA) buffer and frozen. The fraction containing synaptosomes was quantified using Bio-Rad DC protein assay kit.

SUPPLEMENTAL INFORMATION

Supplemental information can be found online at https://doi.org/10.1016/j.stemcr.2023.12.007.

ACKNOWLEDGMENTS

We want to express our gratitude to the patients and their families who generously donated samples to our research. We also extend our thanks to Dr George Chennell, Ms Chen Liang from the Wohl Cellular Imaging Centre, Ms Maeve McLaughlin from the Blizard Institute, and Dr Claire Troakes from the London Neurodegenerative Diseases Brain Bank for their support.

Financial support was received from the Motor Neurone Disease Association (Shaw/Apr15/970-797) and UK Dementia Research Institute (RE11866/PNNTBCR), which receives its funding from UK DRI Ltd., funded by the UK Medical Research Council, Alzheimer's Society, and Alzheimer's Research UK.

AUTHOR CONTRIBUTIONS

Conceptualization, A.L.N. and C.S.; conduct of experiments, A.L.N., C.S., E.C.H., J.A., and N.A.; data analysis, A.L.N. and C.S.; resources, Y.-b.L., G.C., S.C., M.-D.R., and C.E.S.; writing – original draft, C.S. and A.L.N; writing – review & editing, A.L.N., C.S., M.-D.R., and C.E.S.; funding and supervision, C.E.S. and A.L.N.

DECLARATION OF INTERESTS

C.E.S. is the Chief Scientific and Clinical Advisor of AviadoBio.

Received: February 14, 2022
Revised: December 14, 2023
Accepted: December 15, 2023
Published: January 18, 2024

REFERENCES

Barnat, M., Benassy, M.N., Vincensini, L., Soares, S., Fassier, C., Propst, F., Andrieux, A., von Boxberg, Y., and Nothias, F. (2016). The GSK3-MAP1B pathway controls neurite branching and microtubule dynamics. Mol. Cell. Neurosci. *72*, 9–21.

Birsa, N., Ule, A.M., Garone, M.G., Tsang, B., Mattedi, F., Chong, P.A., Humphrey, J., Jarvis, S., Pisiren, M., Wilkins, O.G., et al. (2021). FUS-ALS mutants alter FMRP phase separation equilibrium and impair protein translation. Sci. Adv. *7*, eabf8660.

Blice-Baum, A.C., and Mihailescu, M.R. (2014). Biophysical characterization of G-quadruplex forming FMR1 mRNA and of its interactions with different fragile X mental retardation protein isoforms. RNA *20*, 103–114.

Ceman, S., O'Donnell, W.T., Reed, M., Patton, S., Pohl, J., and Warren, S.T. (2003). Phosphorylation influences the translation state of FMRP-associated polyribosomes. Hum. Mol. Genet. *12*, 3295–3305.

Darnell, J.C., and Klann, E. (2013). The translation of translational control by FMRP: therapeutic targets for FXS. Nat. Neurosci. *16*, 1530–1536.

Darnell, J.C., Van Driesche, S.J., Zhang, C., Hung, K.Y.S., Mele, A., Fraser, C.E., Stone, E.F., Chen, C., Fak, J.J., Chi, S.W., et al. (2011). FMRP stalls ribosomal translocation on mRNAs linked to synaptic function and autism. Cell *146*, 247–261.

Devys, D., Lutz, Y., Rouyer, N., Bellocq, J.P., and Mandel, J.L. (1993). The FMR-1 protein is cytoplasmic, most abundant in neurons and appears normal in carriers of a fragile X premutation. Nat. Genet. *4*, 335–340.

Fujii, R., Okabe, S., Urushido, T., Inoue, K., Yoshimura, A., Tachibana, T., Nishikawa, T., Hicks, G.G., and Takumi, T. (2005). The RNA binding protein TLS is translocated to dendritic spines by mGluR5 activation and regulates spine morphology. Curr. Biol. *15*, 587–593.

Garone, M.G., Birsa, N., Rosito, M., Salaris, F., Mochi, M., de Turris, V., Nair, R.R., Cunningham, T.J., Fisher, E.M.C., Morlando, M., et al. (2021). ALS-related FUS mutations alter axon growth in motoneurons and affect HuD/ELAVL4 and FMRP activity. Commun. Biol. *4*, 1025.

Greenough, W.T., Klintsova, A.Y., Irwin, S.A., Galvez, R., Bates, K.E., and Weiler, I.J. (2001). Synaptic regulation of protein synthesis and the fragile X protein. Proc. Natl. Acad. Sci. USA *98*, 7101–7106.

Hafner, A.S., Donlin-Asp, P.G., Leitch, B., Herzog, E., and Schuman, E.M. (2019). Local protein synthesis is a ubiquitous feature of neuronal pre- and postsynaptic compartments. Science *364*, eaau3644.

He, Q., and Ge, W. (2017). The tandem Agenet domain of fragile X mental retardation protein interacts with FUS. Sci. Rep. *7*, 962.

Imperatore, J.A., McAninch, D.S., Valdez-Sinon, A.N., Bassell, G.J., and Mihailescu, M.R. (2020). FUS Recognizes G Quadruplex Structures Within Neuronal mRNAs. Front. Mol. Biosci. *7*, 6.

Ishigaki, S., Fujioka, Y., Okada, Y., Riku, Y., Udagawa, T., Honda, D., Yokoi, S., Endo, K., Ikenaka, K., Takagi, S., et al. (2017). Altered Tau Isoform Ratio Caused by Loss of FUS and SFPQ Function Leads to FTLD-like Phenotypes. Cell Rep. *18*, 1118–1131.

Jin, P., and Warren, S.T. (2000). Understanding the molecular basis of fragile X syndrome. Hum. Mol. Genet. *9*, 901–908.

Mackenzie, I.R.A., Ansorge, O., Strong, M., Bilbao, J., Zinman, L., Ang, L.C., Baker, M., Stewart, H., Eisen, A., Rademakers, R., and Neumann, M. (2011). Pathological heterogeneity in amyotrophic lateral sclerosis with FUS mutations: two distinct patterns correlating with disease severity and mutation. Acta Neuropathol. *122*, 87–98.

Muddashetty, R.S., Kelić, S., Gross, C., Xu, M., and Bassell, G.J. (2007). Dysregulated metabotropic glutamate receptor-dependent translation of AMPA receptor and postsynaptic density-95 mRNAs at synapses in a mouse model of fragile X syndrome. J. Neurosci. *27*, 5338–5348.

Neumann, M., Roeber, S., Kretzschmar, H.A., Rademakers, R., Baker, M., and Mackenzie, I.R.A. (2009). Abundant FUS-immunoreactive pathology in neuronal intermediate filament inclusion disease. Acta Neuropathol. *118*, 605–616.

Qiu, H., Lee, S., Shang, Y., Wang, W.Y., Au, K.F., Kamiya, S., Barmada, S.J., Finkbeiner, S., Lui, H., Carlton, C.E., et al. (2014). ALS-associated mutation FUS-R521C causes DNA damage and RNA splicing defects. J. Clin. Invest. *124*, 981–999.

Riederer, B.M. (2007). Microtubule-associated protein 1B, a growth-associated and phosphorylated scaffold protein. Brain Res. Bull. *71*, 541–558.

Sahadevan, S., Hembach, K.M., Tantardini, E., Pérez-Berlanga, M., Hruska-Plochan, M., Megat, S., Weber, J., Schwarz, P., Dupuis, L.,

Robinson, M.D., et al. (2021). Synaptic FUS accumulation triggers early misregulation of synaptic RNAs in a mouse model of ALS. Nat. Commun. *12*, 3027.

Salam, S., Tacconelli, S., Smith, B.N., Mitchell, J.C., Glennon, E., Nikolaou, N., Houart, C., and Vance, C. (2021). Identification of a novel interaction of FUS and syntaphilin may explain synaptic and mitochondrial abnormalities caused by ALS mutations. Sci. Rep. *11*, 13613.

Scekic-Zahirovic, J., Sanjuan-Ruiz, I., Kan, V., Megat, S., De Rossi, P., Dieterlé, S., Cassel, R., Jamet, M., Kessler, P., Wiesner, D., et al. (2021). Cytoplasmic FUS triggers early behavioral alterations linked to cortical neuronal hyperactivity and inhibitory synaptic defects. Nat. Commun. *12*, 3028.

Sephton, C.F., Tang, A.A., Kulkarni, A., West, J., Brooks, M., Stubblefield, J.J., Liu, Y., Zhang, M.Q., Green, C.B., Huber, K.M., et al. (2014). Activity-dependent FUS dysregulation disrupts synaptic homeostasis. Proc. Natl. Acad. Sci. USA *111*, E4769–E4778.

Shang, Y., and Huang, E.J. (2016). Mechanisms of FUS mutations in familial amyotrophic lateral sclerosis. Brain Res. *1647*, 65–78.

Telias, M. (2019). Molecular Mechanisms of Synaptic Dysregulation in Fragile X Syndrome and Autism Spectrum Disorders. Front. Mol. Neurosci. *12*, 51.

Vance, C., Rogelj, B., Hortobágyi, T., De Vos, K.J., Nishimura, A.L., Sreedharan, J., Hu, X., Smith, B., Ruddy, D., Wright, P., et al. (2009). Mutations in FUS, an RNA processing protein, cause familial amyotrophic lateral sclerosis type 6. Science *323*, 1208–1211.

Yasuda, K., Zhang, H., Loiselle, D., Haystead, T., Macara, I.G., and Mili, S. (2013). The RNA-binding protein Fus directs translation of localized mRNAs in APC-RNP granules. J. Cell Biol. *203*, 737–746.

Stem Cell Reports, Volume *19*

Supplemental Information

Mutations in FUS lead to synaptic dysregulation in ALS-iPSC derived neurons

Carole Shum, Erin C. Hedges, Joseph Allison, Youn-bok Lee, Natalia Arias, Graham Cocks, Siddharthan Chandran, Marc-David Ruepp, Christopher E. Shaw, and Agnes L. Nishimura

Mutations in FUS lead to synaptic dysregulation in ALS-iPSC derived neurons

Carole Shum [1, 2], Erin C. Hedges [1], Joseph Allison[1], Youn-bok Lee [1], Natalia Arias [1, 3], Graham Cocks [1], Siddharthan Chandran [4], Marc-David Ruepp [1], Christopher E. Shaw [1, 5, 8], and Agnes L. Nishimura [1, 6, 7, 8]

Author affiliations:

[1] United Kingdom Dementia Research Institute Centre, Maurice Wohl Clinical Neuroscience Institute, Institute of Psychiatry, Psychology and Neuroscience, King's College London, 5 Cutcombe Rd, SE5 9RT, London, U.K.

[2] Genetics & Genome Biology Program, The Hospital for Sick Children, Toronto, ON M5G 1X8, Canada; The Centre for Applied Genomics, The Hospital for Sick Children, Toronto, ON M5G 1X8, Canada.

[3] Department of Psychology, Faculty of Life and Natural Sciences, Brain and Behavior Group, Nebrija University, Madrid, Spain.

[4] MRC Centre for Regenerative Medicine, Euan MacDonald Centre for MND Research and Centre for Clinical Brain Sciences, University of Edinburgh, Edinburgh, EH16 4SB, UK

[5]Centre for Brain Research, University of Auckland, 85 Park Road, Grafton Auckland 1023, New Zealand

[6] Centre for Neuroscience, Surgery and Trauma, Blizard Institute, Barts and The London School of Medicine and Dentistry, Queen Mary University of London, London, UK

[7] Institute Paulo Gontijo, Brazil

Author footnotes: 8 These authors contributed equally

Contact information: Dr Agnes Nishimura (a.nishimura@qmul.ac.uk) and Prof. Christopher E. Shaw (chris.shaw@kcl.ac.uk)

Supplementary experimental procedures

Human primary cell lines

Two FUS fibroblast lines were established from a skin biopsy taken from a female carrying the R521C FUS mutation and a male patient carrying the R514G mutation. The biopsy was cut into pieces, plated in a tissue culture flask, and grown in a human fibroblast derivation medium containing DMEM, 10% fetal bovine serum, and 1% penicillin/streptomycin. The outgrowth of cells appeared after 1-2 weeks. The medium was replaced every two days, and cells were passaged 1:3 with 0.25% trypsin-EDTA upon reaching confluency. All procedures were approved by the King's College Hospital, NHS Foundation Trust ethics review committee and the Department of Research and Development.

Generation and expansion of iPSCs

Induced pluripotent stem cells were reprogrammed from dermal fibroblasts, keratinocytes and lymphoblastoid cell lines as previously reported (Bilican et al., 2012; Rajesh et al., 2011). For fibroblast-derived iPSCs, approximately 1 x 10^5 control and patient cells were plated in one well of a six-well plate and infected with the retrovirus expressing OCT4, NANOG, c-MYC and SOX2 as described previously (Barrett et al., 2014; Bilican *et al.*, 2012). Three days post-infection, cells were passaged into plates pre-seeded with inactivated mouse embryonic fibroblasts (MEFs). Seven days after infection, the medium was transitioned to human embryonic stem cell (hESC) medium (DMEM/F12 containing 20% Knockout Serum Replacement, ten ng/mL human recombinant basic fibroblast growth factor (bFGF), 1 x non-essential amino acids, 5.5 mM β-Mercaptoethanol (Sigma Aldrich) and 1 x penicillin-streptomycin), supplemented with five µM ROCK inhibitor (Y27632; Calbiochem) until visible colonies appear. Multiple colonies were collected and expanded. Lymphoblastoid derived iPSCs were generated by transfecting 1x10^6 cells with plasmids expressing OCT4, L-MYC, KLF4, SV40LT, LIN28, SOX2 and shRNA-p53 (Barrett *et al.*, 2014). Cells were plated on MEF until visible colonies appear. Colonies were manually picked and expanded. All iPSCs lines were cultured in E8 Flex media in feeder-free conditions until differentiation.

Lentiviral production

The glutamate experiments were performed in neurons transduced with FU:MAP2-GFP plasmid for better visualisation of varicose dendrites and axons. EGFP sequence was subcloned into FU-MAP2-Gateway plasmid (a gift from John Gearhart (Addgene plasmid # 43915; **http://n2t.net/addgene:43915**; RRID:Addgene_43915). Lentiviral particles were produced in HEK293FT in-house and stored at –70°C until use.

Quantitative PCR

RNA was isolated with the RNeasy kit Plus (Qiagen) according to the manufacturer's instructions. cDNA was synthesised by using 1 µg of total RNA using iScript cDNA synthesis kit (BIO-RAD) and quantitative PCR was performed using PowerUP SYBR green master mix following manufacturer's instructions. qPCR reactions were amplified in QuantStudio 7 Flex Real-Time PCR system (Applied Biosystems) and analysed in QuantStudio Real-time PCR system software (Applied Biosystems). Primer sequences for FMR1: forward: 5'-GGAACAAAGGACAGCATCGC-3'; reverse:

5’-CTCTCCAAACGCAACTGGTCT-3. Primer efficiency (E) value was calculated for FMR1 expression based on the slope of the standard curve. The E value for FMR1 expression is 2.06, and this value was incorporated in the formula:

$$RQ = \frac{E^{\Delta CT\ GOI}}{average\ ERef^{\Delta CTRef}}$$

Where:

RQ = relative quantity

E=primer efficiency

∆CT= control Ct– FUS Ct

GOI=Gene of interest

Ref=housekeeping genes

Glutamate toxicity

Neurons were plated on 96 well plates for two weeks and transduced with FU:MAP2-GFP lentivirus, following another two weeks in culture. Cells were treated with vehicle (DMSO) or 100 mM Glutamate (Abcam) for 24 hours and fixed. Cells were stained for caspase-3 antibody (BD Biosciences) and imaged using the Opera Phenix High Content Screening microscope. The total number of neurons analysed was control 1 (n=1,003), control 2 (n=1,206), R514G (n=1,634) and R521C (n=1,205).

Immunostaining

Cells were fixed with 4% paraformaldehyde or with 4% paraformaldehyde for 10 min at room temperature followed by incubation in methanol pre-chilled to -20°C for 10min, permeabilised with 0.5% Triton X-100 for 15 minutes at room temperature, then blocked in 10% donkey serum for one hour at room temperature, followed by incubation with primary and secondary (Dylight dyes) antibodies. The nuclei were counterstained with DAPI staining and coverslips mounted on slides with mounting media (DAKO).

Calcium imaging and synaptic activity analyses

Cortical neurons were plated in 96 well plates (Falcon) and aged for 108 days. Cells were incubated with 2 mM Fluo4-AM in external solution (145 mM NaCl, 2 mM KCl, 5 mM $NaHCO_3$, 1 mM $MgCl_2$, 2.5 mM $CaCl_2$, 10 mM glucose, 10mM Na-HEPES pH 7.25) or FluoroBrite DMEM media (Life Technologies) and 0.02% Pluronic-F27 for 15 mins at 37°C. Subsequently, neurons were rinsed in external solution for another 15 mins at 37°C. 80 frames were taken every second and processed in ImageJ. Spontaneous calcium fluctuation was calculated as relative Fluo4-AM fluorescence intensities compared to background: F-F0/F0. Imaging was performed in the Opera Phenix Content Screening microscope (20x water objective). 10 regions of interest (ROI) were measured and plotted on a graph to represent calcium fluctuation during time.

To determine whether calcium influx is altered upon depolarisation, 4 weeks old neurons were treated with 2 μM Fluo4-AM in FluoroBrite DMEM media and incubated for 15 mins at 37°C, followed by 15 mins wash with FluoroBrite DMEM media. Cells were imaged for 60 seconds to calculate basal fluorescence and 100 mM KCL was added to the media. Imaging recording was performed for 300 seconds. 5-10 ROIs were selected, and fluorescence was measured over time.

To measure synaptic activity, cortical neurons were cultured for 4 weeks and treated with 10 μM of FM4-64. FM4-64 incorporates into the membrane and is internalised by endocytosis when becomes fluorescent. At synapses, FM4-64 is in the synaptic vesicle membrane and after depolarisation of the membrane with KCL, the synaptic vesicles are released into the synaptic cleft, losing the fluorescence.

Western blot

Total extracts were generated from neurons by lysing the cells in 1X cold RIPA lysis buffer (50 mM Tris-HCl (pH7.4), 150mM NaCl, 1% (v/v) Triton X-100, 0.1% (w/v) sodium dodecyl sulfate (SDS), 0.1% (w/v) Sodium Deoxycholate), supplemented with 1x Proteinase inhibitor (Roche) 1x Phosphate inhibitor (Roche). Cells were incubated on ice for 30 minutes, followed by sonication for 20 seconds. Cellular lysate was centrifuged at 14,000 x g at 4°C for 20 minutes. Protein concentration was measured using DC Protein assay (BIO-RAD) and 1x SDS sample buffer (5x SDS sample buffer containing 10% (w/v) SDS, 250 mM Tris-HCl (pH 6.8), 50% (v/v) glycerol, 500mM (w/v) dithiothreitol, 0.25% (w/v) bromophenol blue) was added in each sample. Approximately 10 µg of protein was loaded in 10% NuPAGE Bis-Tris precast gels and transferred onto nitrocellulose membranes using iBlot2 Gel transfer device. Membranes were blocked with 5% BSA or non-fat milk, followed by incubation of primary antibody in 1% BSA or non-fat milk overnight at 4°C. Membranes were rinsed with TBST, followed by incubation of secondary antibodies in the same buffer. Membranes were developed using Odyssey CLx Imager (Li-CoR Biosciences).

One microliter of protein weight standards (Precision Plus Protein Dual Color Standards, BIO-RAD) was loaded in each gel for protein molecular weight estimation.

Fluorescence In situ Hybridisation

Cortical Neurons were cultured for six weeks, fixed with 4% paraformaldehyde for 15 minutes at room temperature, and pre-treated with 70% ethanol for 2 mins at -20°C. Coverslips were stored in 100% ethanol at -80°C until staining. On the day of fixing, ethanol was removed, and coverslips were incubated with 70% ethanol for 2 mins at -20°C, followed by three washes of PBS for 5 mins each.

Cells were blocked with 1% BSA in PBS and 200 mM ribonucleoside vanadyl complex (RVC) for 30 mins at room temperature. Cells were washed twice with 2× SSC (300 mM NaCl, 30 mM sodium citrate pH 7.0) and incubated in **pre-hybridization buffer** (15% formamide, 10 mM sodium phosphate, 2× SSC) for 10 min at room temperature.

Prepare 40nM *FMR1* probe in hybridisation buffer (10% dextran sulfate, 2mM RVC, 2XSCC, 10 mM sodium phosphate, 15% Formamide, 0.2% BSA 0.5 mg/ml salmon sperm) and boil at 80°C for 5 mins and transfer to ice. Add the probe to the cells and incubate at 37°C overnight in a wet chamber to avoid evaporation.

On the next day, wash cells with pre-hybridisation buffer for 30 mins at 37°C twice, followed by two washes of a solution containing 20% formamide, 2mM RVC and 0.05XSSC for 10 mins. A final wash of 2XSSC followed this for 2 mins at room temperature.

The coverslips were mounted and imaged using the INCell Analyser 6000 and Zeiss LSM 880.

The *FMR1* probe (GGACUUGUUUUUGUUUUUGUUUUGUUGCACUGAAGUUUGAUA) was obtained from Integrated DNA technologies.

GST pulldown

GST, GST-FUS wt, GST-FUS R521C, GST-FUS R514G, GST-FUS-∆RRG3 (1-456 aa) and GST-FUS-RRG3 (423-526) constructs were generated and transformed into BL21 competent bacteria. Single bacteria clones were inoculated in Luria broth media containing 100 mg/ml ampicillin overnight at 37°C in constant agitation. The next day, 10% of a starter culture was transferred to a new tube containing Terrific broth and kept in agitation at 37°C until OD 0.6-0.8 when 0.1 mM of IPTG was added to induce expression for another 4 hours. Bacteria were pelleted at 4,000 x g for 10 mins at room temperature and frozen at -80°C until use.

Lysis buffer was added to the bacteria pellet following the MagneGST protein Purification system (Promega) instructions.

HEK cells were transfected with eGFP-FMRP construct using lipofectamine 2000. Approximately 10 mg of HEK cell lysate was incubated to 1 mg of GST construct with or without RNAse, following the MagneGST protein Purification system (Promega) instructions. The final magnetic bead was boiled in 1X laemmli buffer (BioRad) and resolved in western blots.The membranes were incubated with FUS (Proteintech) antibody and FMRP (Cell Signaling Technology).

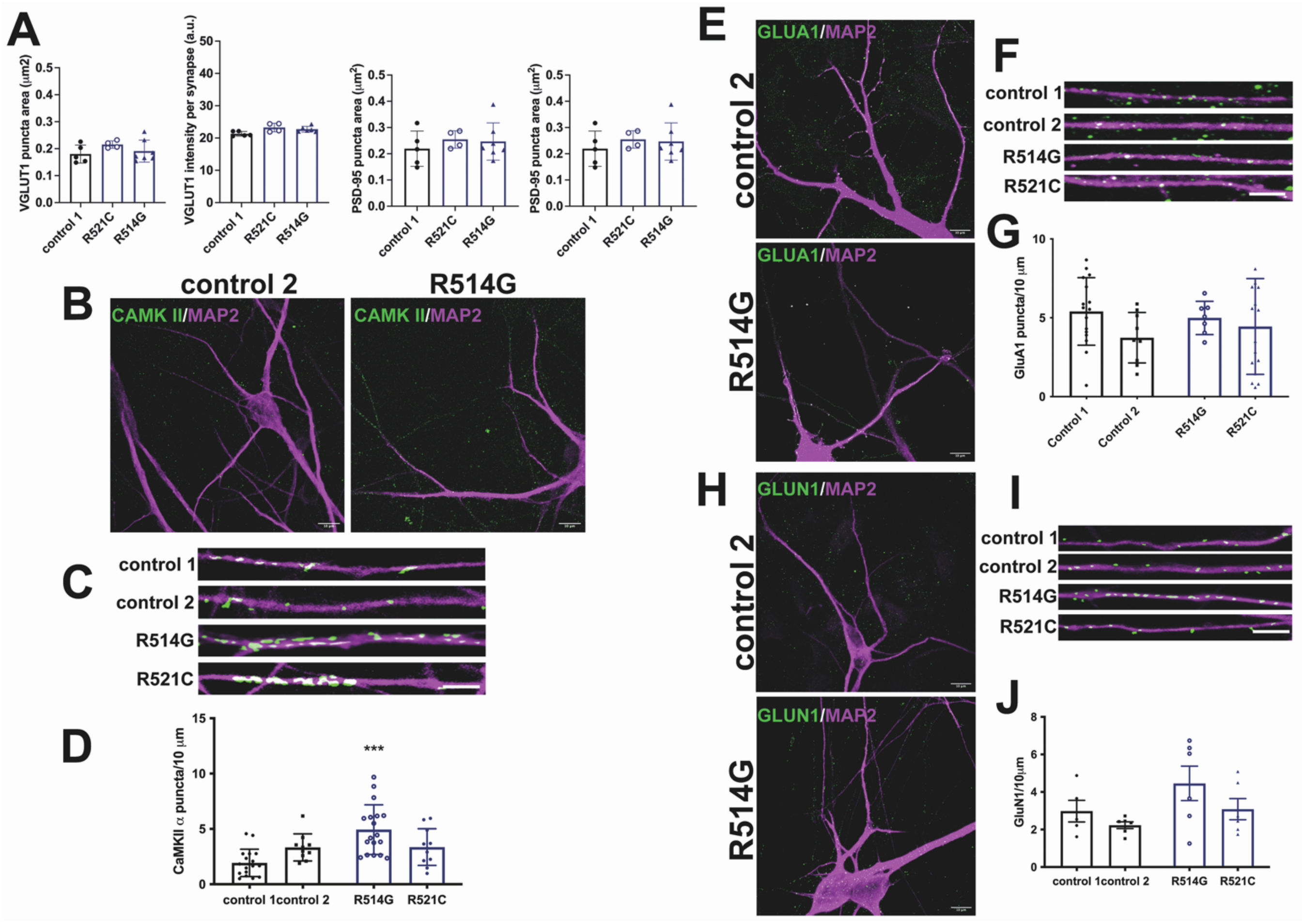

Supplementary Fig 1. **A)** Quantification of PSD-95 and VGLUT1 area and intensity from colocalised puncta. Data represent neurons analysed per group from three biological replicates**. B)** Representative confocal image of neurons expressing CAMKIIa. **C)** Representative dendrites showing CAMKIIa puncta. Scale bar = 10 μm. **D)** Quantification of CAMKIIa puncta per 10 μm dendrite. Data points represent single neurons measured as mean ± SD of 3 independent experimental replicates. **E)** Representative confocal image of neurons expressing the ionotropic glutamate receptor AMPA type GLUA1. Scale bar = 10 μm. **F)** Representative dendrites showing GLUA1 puncta. **G)** Quantification of GLUA1 puncta per 10 μm dendrite. No significant difference was observed between the two groups. Data points represent single neurons measured as mean ± SD of 3 independent experimental replicates. **H)** Representative confocal image of neurons expressing the glutamate receptor NMDA type GLUN1. **I)** Representative dendrites showing GLUN1 puncta. Scale bar = 10 μm. **J)** Quantification of GLUN1 puncta per 10 μm dendrite. Data points represent single neurons measured as mean ± SD of 3 independent experimental replicates.

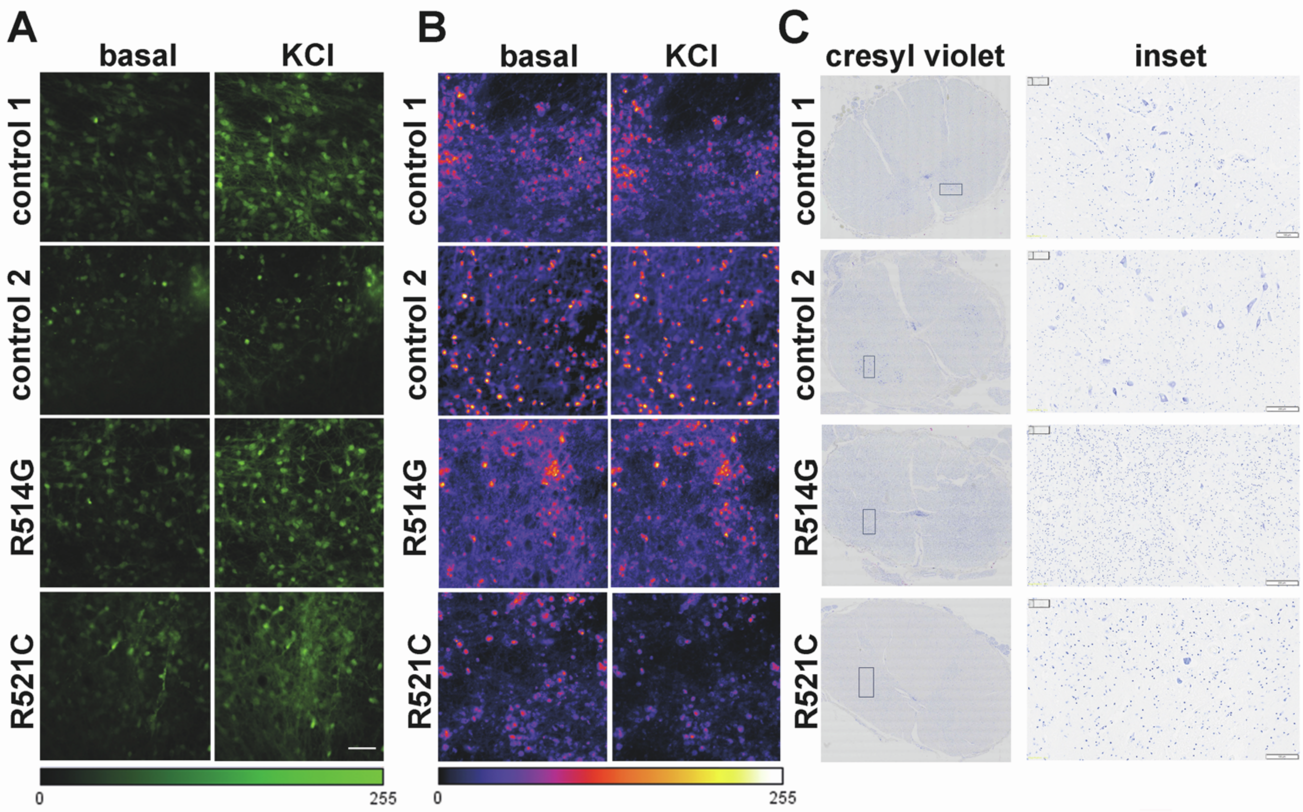

Supplementary Fig. 2. A) Representative images of neurons after Fluo-4-AM calcium staining in basal levels and after KCl depolarisation. Scale bar = 50 μm. **B)** Representative images of neurons after FM4-64 staining in basal levels and after KCl depolarisation. Scale bar = 50 μm. **C)** Cresyl Violet staining of post-mortem sections of spinal cord of control and FUS-ALS patients. A drastic reduction of motor neurons is observed in the FUS-ALS cases. Scale bar 100 μm.

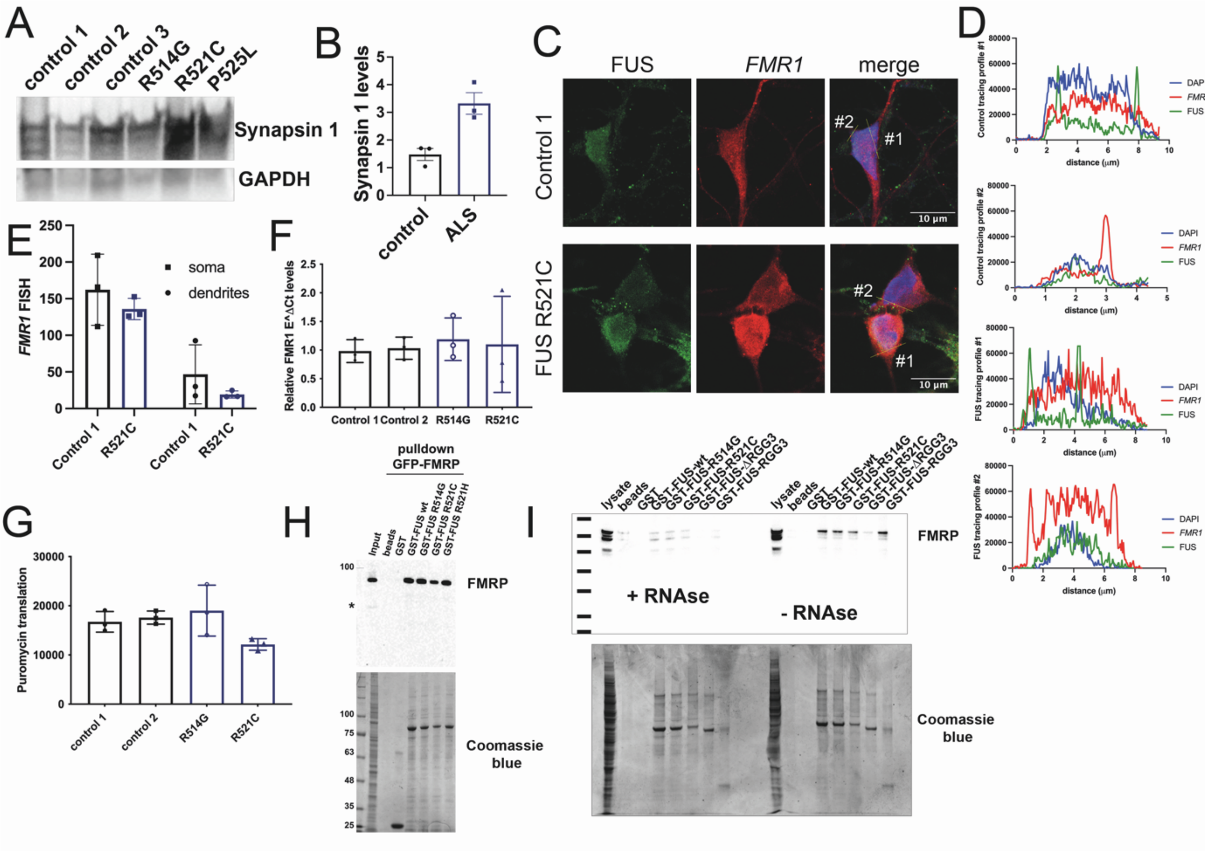

Supplementary Fig 3. A) Western blot analyses of synaptosome fraction from post-mortem cerebral cortex samples showing an increase of Synapsin 1 in FUS-ALS patients and relative expression of Synapsin 1. B) Quantification of relative Synapsin I levels as in A. C) Fluorescent In situ hybridisation (FISH) of *FMR1* mRNA indicates *FMR1* is not recruited into FUS granules in the soma or neurites. Representative images of neurons stained for FUS and *FMR1*. D) Line profile from two regions of interest. Note that FUS does not colocalise with FMR1 granules and vice-versa. A slight decrease in *FMR1* levels was observed. E) Quantification of *FMR1* FISH. F) Relative total *FMR1* q-PCR data. Data points represent three independent experiment replicates. G) Puromycin translation in FUS-ALS and control iPSC-derived neurons. Data points represent three independent experimental replicates. H) GST pulldown shows direct binding of FUS and FMRP. *Endogenous FMRP. GST proteins were incubated with eGFP-FMRP proteins and membranes were incubated with FMRP antibody. I) GST pulldown of FUS and FMRP is mediated by RNA. Note that the RGG3 domain containing the NLS regions is essential for FUS-FMRP binding.

Supplementary Table I: Clinical information for all ALS and control post-mortem spinal cord samples.

	age	sex	PMD	DIAG	Fz area	mutation
**SC-01**	60	M	66	MND	T	R514G
**SC-02**	23	F	37	MND	T	P525L
**SC-03**	39	M	51	MND	L	K510E
**SC-04**	34	F	38	MND	L	R495X
**SC-05**	35	F	19	fMND	L	R521H
**SC-06**	35	F	24	fMND	L	R521C
**SC-07**	33	M	71	fMND	L	R521C
**SC-08**	40	M	40	Control	L	-
**SC-09**	51	F	33	Control	L	-
**SC-10**	54	M	30.5	Control	L	-
**SC-11**	55	F	95	Control	L	-
**SC-14**	63	M	23	Control	L	-

References

Barrett, R., Ornelas, L., Yeager, N., Mandefro, B., Sahabian, A., Lenaeus, L., Targan, S.R., Svendsen, C.N., and Sareen, D. (2014). Reliable generation of induced pluripotent stem cells from human lymphoblastoid cell lines. Stem Cells Transl Med *3*, 1429-1434. 10.5966/sctm.2014-0121.

Bilican, B., Serio, A., Barmada, S.J., Nishimura, A.L., Sullivan, G.J., Carrasco, M., Phatnani, H.P., Puddifoot, C.A., Story, D., Fletcher, J., et al. (2012). Mutant induced pluripotent stem cell lines recapitulate aspects of TDP-43 proteinopathies and reveal cell-specific vulnerability. Proc Natl Acad Sci U S A *109*, 5803-5808. 10.1073/pnas.1202922109.

Rajesh, D., Dickerson, S.J., Yu, J., Brown, M.E., Thomson, J.A., and Seay, N.J. (2011). Human lymphoblastoid B-cell lines reprogrammed to EBV-free induced pluripotent stem cells. Blood *118*, 1797-1800. 10.1182/blood-2011-01-332064.
